# Always Getting Lost: Defining Developmental Topographical Disorientation (DTD)—A Systematic Literature Review

**DOI:** 10.1007/s11065-025-09664-8

**Published:** 2025-05-14

**Authors:** Ineke J. M. van der Ham, Michiel H. G. Claessen

**Affiliations:** Department of Health, Medical and Neuropsychology, Faculty of Social Sciences, Leiden University, Wassenaarseweg, 52, 2333 Leiden, AK the Netherlands

**Keywords:** Developmental topographical disorientation, Spatial representation, Mental map, Spatial navigation, Landmark knowledge

## Abstract

Developmental topographical disorientation (DTD) refers to a condition of highly impaired navigation ability in healthy individuals. DTD often leads to severe consequences in daily life, affecting education and professional choices and limited everyday mobility. Since its first description in 2009, a substantial number of empirical studies on DTD have appeared, but a clear clinical definition of DTD that can be used to develop a behavioral assessment tool is not yet available. The aim of the current study was to shed more light on the precise behavioral characteristics of DTD by examining the empirical evidence available to date. Recent theoretical developments that enable the classification of navigation impairment in various populations are utilized in the current work. Through a systematic literature review, reported descriptions and criteria for DTD were identified. Furthermore, tests included and performance of people with DTD are classified in the different navigation domains relevant to navigation impairment (landmark knowledge; location knowledge, egocentric and allocentric; and path knowledge, route and survey). A total of 15 empirical papers were included in the analyses, each discussing performance of people with DTD in large-scale spatial tasks. Initial DTD descriptions focused on mental map quality, whereas later work adheres to a more general definition of impaired navigation. Performance patterns show that the navigation impairment in DTD is largely attributable to low mental map quality, as low performance is primarily found for tasks measuring allocentric location knowledge and path knowledge. In contrast, landmark knowledge remains largely unaffected and, if impaired, appears to also include face recognition impairment, suggesting a more general form of visual agnosia. Egocentric location knowledge is often not included in assessments. The outcomes support the initial focus on poor mental map quality as the key characteristic of DTD, combined with a landmark-focused navigation strategy. The current findings therefore provide relevant input to the development of a clinical characterization of DTD and the development of appropriate assessment tools.

## Introduction

Successful spatial functioning is an essential element of nearly all daily life activities. Large-scale spatial abilities help us plan routes and navigate to important locations that allows us to shop for food, interact with social contacts, and go to our place of work or education (see e.g. Denis & Loomis, 2007; Epstein et al., 2017). Whereas getting lost can be a frequent observation after acquired brain injury (see e.g. Claessen & van der Ham, 2017), in healthy population, spatial navigation generally is at a sufficient level. However, there are exceptional cases in which spatial navigation is severely limited, or even absent, even within healthy population. The condition of developmental topographical disorientation (DTD) was first named in 2009 by Giuseppe Iaria and colleagues. According to more recent theoretical work on DTD (e.g. Iaria, 2013; Iaria & Burles, 2016), its criteria are fourfold: (1) getting lost at least multiple times a week; (2) the problem of getting lost has been present since childhood; (3) there are no other noticeable cognitive problems that could explain for getting lost; and (4) there are no neurological or psychiatric abnormalities that could explain for getting lost. DTD has especially surfaced due to fascinating case descriptions of people who are unable to use any kind of spatial information to travel by themselves, leading to severe limitations in educational and professional activities. As standardized testing of navigation ability is hardly available (see e.g. van der Ham et al., 2013; van der Ham & Claessen, 2022), most cognitive measures that have been used so far focus on learning routes in known and unknown environments and drawing maps of familiar environments such as their own house (see e.g. Iaria et al., 2009; Iaria & Barton, 2010). In case of severe DTD, such maps are typically devoid of spatial detail and show that only the order of rooms can be represented, but that distinctions in shape, proportion, and relative position are absent (e.g. Bianchini et al., 2014; Iaria et al., 2009). When Iaria and colleagues attracted public attention to this newfound condition, they were able to conduct several group studies, to provide information of the condition, and to initiate interaction between people with DTD. From personal stories, it shows that people with DTD can experience social-emotional difficulties due to their condition (Iaria & Burles, 2016), such as self-esteem problems and limitations in education. Similar social-emotional difficulties have been found, comparable to those found for dyslexia (see e.g. Riddick, 2000; Shaw & Anderson, 2017; Stoeber & Rountree, 2021). DTD can be mistaken for being immature, too dependent or anxious, and be perceived as a sign of low intelligence. In addition, people with DTD may be forced to limit their educational and professional options, simply because they are not able to attend the relevant locations independently. Thus, in some cases, limited intellectual development might be a consequence of DTD, rather than a cause.

With the literature available at this point, several characteristics of DTD can clearly be identified. It concerns a problem with spatial navigation since birth, lacking alternative explanations concerning more general cognitive or mental health problems, there appears to be a genetic component (Barclay et al., 2016), and its prevalence in healthy population is estimated at 3% (Piccardi et al., 2022a, 2022b). There are also some elements that remain unclear. A precise definition in terms of cognitive characteristics is lacking. Some argue that any developmental problem concerning navigation can be considered DTD, including landmark agnosia (Piccardi et al., 2019), whereas others suggest a specific role for mental mapping and its neural correlates—hippocampal formation—specifically (e.g. Conson et al., 2018; Iaria et al., 2014). Resolving the inconsistencies in these distinct descriptions of DTD is a pivotal step towards its clinical classification. Furthermore, substantial variation exists between individual cases of DTD, where the individual case studies could possibly reflect the more extreme examples and therefore provide a somewhat biased perspective of DTD (as compared to a large group study by Burles and Iaria (2020)). Variation in findings is further caused by the variation in the tasks used, which can differ between the different labs performing the assessments. This is understandable as standardized testing materials for large-scale spatial functioning have long been absent and are only recently being made available (e.g., virtual SILCton, Weisberg et al., 2014; Sea Hero Quest, Spiers et al., 2023; and Leiden Navigation Test, van der Ham & Claessen, 2022). Another factor that may hinder the diagnostic process of DTD, is the emergence of navigation assistance options since the first report on DTD. Currently, many people have technological assistance available to them through smartphones and car navigation systems. From our discussions with people with DTD, we observed that the question “how frequently do you get lost” might be difficult to answer, as many of them indicate their answer would depend on if they would have access to their phones or not. Such technology could thus mask DTD to some extent, and for some provide some support.

The current state of research on DTD brings forward two primary issues: that of potential fallacies at hand, and concerns with regard to construct validity. As recently discussed by Hanfstingl et al. (2024), it could be that DTD suffers from a jingle fallacy (Thorndike, 1904), in which one term—in this case DTD—is used to describe distinct phenomena. Because a detailed cognitive characterization of DTD is currently lacking, there is a risk that all descriptions of people experiencing a specific weakness in navigation behavior are covered by DTD, while they may not all reflect the same behavioral phenomenon. The process suggested by Hanfstingle and colleagues to unravel a jingle fallacy is to reach reconceptualization by examining existing theory and data. Currently, the construct validity of DTD measures is low, as they rely heavily on self-report and suffer from a lack of standardized testing materials concerning navigation behavior.

The intention of the current systematic literature review is therefore to explore how DTD could be defined in a more objective manner, resolving the jingle fallacy and providing the foundation for a formal assessment procedure to be developed, with sufficient construct validity. If in future work a formal test could be developed based on the characterization of DTD, this would then be helpful in offering perspective to those with DTD: if standardized testing is available, stigma could be relieved and specialized support systems could be developed. Our previous work on objective classification of navigation impairment in individuals with acquired brain injury can be highly informative to the identification of DTD as well (e.g. van der Kuil et al., 2022). Following Claessen and van der Ham (2017), navigation impairment can occur in three different forms: landmark knowledge; location knowledge, either egocentric or allocentric; and path knowledge, either based on route or survey information. Landmark knowledge refers to the ability to memorize and recognize distinct landmarks or scenes in an environment. Location knowledge relates to the spatial locations of these landmarks, which can be assessed in either an egocentric or an allocentric manner (e.g. Burgess, 2006; Klatzky, 1998). Egocentric perspective taking in navigation refers to observer-based encoding of spatial information, which can be assessed by asking to point towards specific locations (e.g. Wang & Spelke, 2000). In contrast, allocentric perspective taking involves environment-based encoding of spatial locations, measured by tasks in which locations are encoded based on external spatial cues (Thornberry et al., 2021). Path knowledge refers to knowledge concerning the spatial location of multiple landmarks in an environment, which can either be used in a dynamic manner (e.g., memory of route elements like turns and sequences) (e.g. Wolbers et al., 2004) or a static manner (e.g., survey knowledge through distance comparisons of different pairs of landmarks) (Chrastil & Warren, 2013).

Here, we aim to examine existing empirical literature on DTD and study what is thus far known about cognitive performance in DTD. To this end, we conducted a comprehensive literature search on any scientific publications that focus on behavioral measures of “developmental topographical disorientation.” Our main aim was to attempt to specify DTD in more detail based on existing scientific evidence, keeping the different domains of navigation ability, as described above in mind. Such specification can be considered the theoretical and empirical verification of the potential jingle fallacy currently present for DTD; what is the precise and unique phenomenon of DTD? Based on the outcome of this search, we first gathered the commonly used descriptions for DTD. Next, we analyzed the specific cognitive tests and questionnaires used in populations with DTD and their performance on these measures, to clarify which cognitive functions show atypical characteristics, which functions are generally intact, and which potentially relevant functions have not been examined yet. In particular, the tests were characterized according to the different navigation domains (landmark; location, egocentric and allocentric; path, route; path, survey; Claessen & van der Ham, 2017), to examine whether there may be a specific pattern of impairment that can be linked to DTD. As many case studies on DTD are illustrated with indirect behavioral expressions such as severely impaired map drawings of even very familiar environments, such as someone’s own house, we hypothesized that DTD relies heavily on impaired mental map formation and use. This is also in line with recent evidence of atypical connectivity patterns in the hippocampal region, related to DTD (e.g. Fragueiro et al., 2024; Iaria et al., 2014; Kim et al., 2015). In terms of navigation domains, this would mean that primarily tasks measuring allocentric location and path survey knowledge would be impaired, as mental map use is most relevant to these domains. The lists of included cognitive functions will also be analyzed to identify which cognitive functions may need further evaluation in future work. Lastly, additional qualitative observations made during the analyses of the set of articles will be presented and discussed.

## Methods

### Search Procedure

On 12 January 2024, a systematic literature search was performed following the PRISMA guidelines, using three databases: PubMed, PsychINFO, and Web of Science. The review was not registered. As we were specifically interested in DTD, the search terms used were relatively simple. We used (developmental topographical disorientation) as the search terms, with filters for English (all three search engines), human (PubMed and PsychINFO), and full text (PubMed), with no limitation on publication date. Inclusion criteria for the title, abstract, and full text searches were (1) the main topic of the paper is DTD, (2) only healthy participants with no other cognitive or physical impairments that could possibly explain for navigation impairment were studied, and (3) the paper included the presentation of behavioral data of large-scale spatial functioning.

**Fig. 1:**
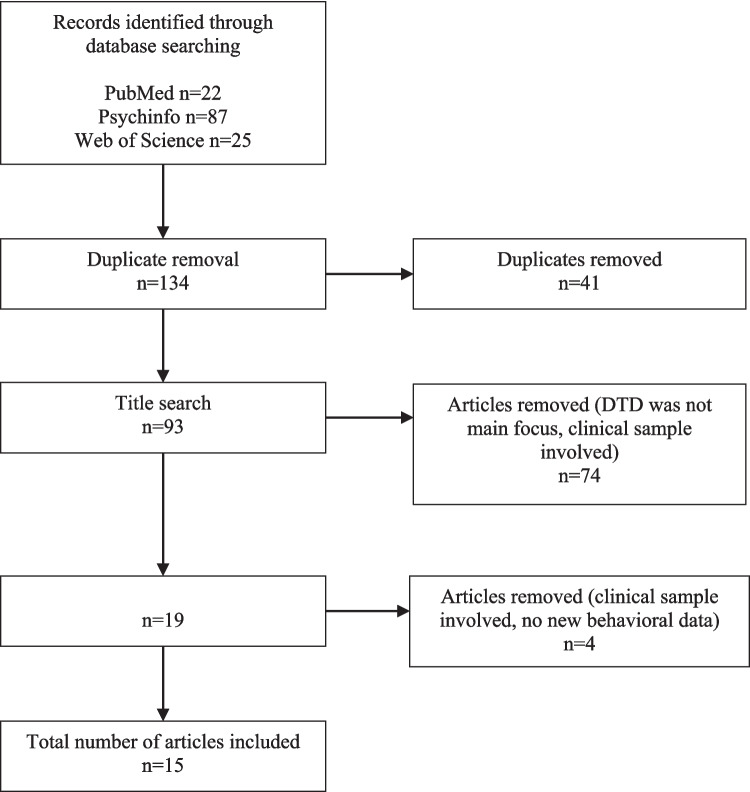
Flowchart depicting the literature selection process

For DTD, a total of 93 hits were found, after duplicate removal (41 removed). In Fig. 1, the flowchart of the approach is depicted. The search was performed by three independent researchers. During the title search, 74 articles were excluded, due to absence of DTD as the primary focus, or when it was clear there was a focus on clinical rather than healthy participants. Of the remaining 19 articles, another four were excluded during the abstract search due to participants with clinical conditions, or the absence of new behavioral data, resulting in a final dataset of 15 articles.

### Analyses

For the analyses, the focus was twofold: (1) to understand the defining features of DTD as used by the authors and (2) to examine the cognitive characteristics of the tasks used and the performance patterns of the participants. All introduction and methods sections were analyzed by three independent researchers to extract the criteria used for DTD. All methods sections were processed to extract task characteristics and to identify the cognitive constructs tested in each task and the characteristics of the sample of participants. Through examination of all results sections, each cognitive construct measured was marked as being intact or impaired in comparison to healthy control participants, as indicated by the authors.

## Results

In total, 15 articles have addressed DTD in an experimental setting (see Table 1). Eight of these articles concern single case studies; the remaining seven articles included between two and 1211 participants with DTD.

**Table 1:** List of included articles, with total *n*, gender distribution and (mean) age of DTD participants, definition of DTD used in the article (quoted), and comorbidities reported

Article	*n*	Gender	Age	Definition DTD	Comorbidities
Iaria et al., 2009	1	F	43	Deficit in acquisition of mental representation	-
Bianchini et al., 2010	1	M	22	Deficit in segregating landmarks and deriving navigational information from them, together with the absence of any non-verbal navigational process, and the presence of a deficit in generating cognitive maps	-
Iaria & Barton, 2010	120	102 F	43.7	Selective impairment in the ability to form a mental representation of the environment that is a cognitive map	-
Iaria et al., 2014	9	F	52.67	(a) Getting lost frequently in very familiar surroundings, (b) experiencing the problem since very familiar surroundings, (c) having no other cognitive complaints affecting daily life activities, and (d) having not suffered any brain injury daily life activities	Face recognition impairment in six individuals
Palermo et al., 2014a, 2014b	2	M	29 and 38	DTD is a selective difficulty in orienting and wayfinding showed by individuals who never developed normal topographical competencies despite their otherwise typical cognitive and intellectual development, their average (or above average) IQ, and the absence of any neurological or psychiatric disorder	-
Palermo et al., 2014a, 2014b	1	M	38	The presence of navigational difficulties in the context of normal intellectual ability and in the absence of any known perinatal, neurological, or psychiatric disorders	-
Bianchini et al., 2014	1	M	29	A selective disorder of topographical orientation and navigation in people who have no cerebral damage or perinatal problems	-
Nemmi et al., 2015	1	M	28	Different deficits involving specific modules of the navigational system	-
Barclay et al., 2016	16	15 F	58.3	See Iaria et al. (2014)	-
Conson et al., 2018	1	F	20	Substantial difficulties in building up mental representations	-
Piccardi et al., 2019	1	M	6	Individuals who never developed the capability to move through the environment	Prosopagnosia
Burles & Iaria, 2020	1211	1015 F	35.66	See Iaria et al. (2014)	Lower imagery related to objects/faces/places
Rusconi et al., 2021	1	F	22	Not mentioned	Face recognition deficit
Piccardi et al., 2022a	54	11 F	24.8	Low score on Sense of Direction scale	-
Piccardi et al., 2022b	54	11 F	24.8	Low score on Sense of Direction scale	-

### Descriptions of DTD

The first use of the term developmental topographical disorientation is relatively recent and stems from the work of Giuseppe Iaria and colleagues from 2009 onwards. Notably, our search showed that Brunsdon, Nickels, Coltheart, and Joy (2007) also mention a developmental type of topographical disorientation, to refer to substantial difficulty in navigation in a child. In their case description, visual agnosia was also present and therefore not included in our current analyses. Since 2009, an additional 14 experimental articles have been published, all included in the analyses reported here. In the article by Iaria and colleagues (2009) first mentioning DTD, the condition is described as a deficit in the acquisition of mental representation. An explicit reference to mental or cognitive map quality is referred to by the two oldest publications after the initial work (Bianchini et al., 2010; Iaria & Barton, 2010) and Conson and colleagues (2018). Starting in 2014, Iaria and colleagues (2014) explicitly propose four criteria for DTD: (1) getting lost frequently in very familiar surroundings, (2) experiencing the problem since childhood, (3) having no other cognitive complaints affecting daily life activities, and (4) having not suffered any brain injury or neurological conditions. These criteria are referring to navigation inability in a general sense, rather than specifying mental map quality. In most of the newer articles, these criteria are used as well. Nemmi and colleagues (2014) explicitly highlight that given the complexity of navigation ability, DTD can stem from various forms of navigation impairment, related to the different “modules” of navigation that can be selectively impaired. Piccardi and colleagues (2019) have taken the idea of diversity within DTD further by introducing a case of Developmental Landmark Agnosia as a subtype of DTD. Notably, this case was the only child reported in all articles included (6 years old, the next youngest participant was 20 years old, described by Conson et al. (2018)), and the data show that prosopagnosia was also observed in combination with landmark agnosia. This link to face processing, along with potential subtypes of DTD, is also considered by Rusconi and colleagues (2021). The two newest articles in our search result use a markedly different way to determine presence of DTD. Piccardi and colleagues (2022a, 2022b) focus on a sense of direction measure to extract people with DTD from a large population wide study. A sense of direction score of more than two standard deviations below the mean of the sample is considered a classifier for DTD.

Furthermore, the definitions for the phenomenon of DTD showed informative patterns. Most of the articles do not indicate comorbidity with other forms of cognitive impairment (12 out of 15). In three of the articles, prosopagnosia or markedly lower performance in face recognition is reported in relation to DTD.

### Cognitive Measurements

For each of the articles, all behavioral measures were analyzed and categorized according to the spatial cognitive characteristics. Analogous to previous work on navigation impairment (Claessen & van der Ham, 2017; van der Ham & Claessen, 2020; van der Ham et al., 2020), five categories were used: landmark knowledge (identifying landmarks or scenes of an environment), egocentric location knowledge (directional coding of specific locations in an environment, e.g., pointing), allocentric location knowledge (environment-based coding of specific locations, e.g., indicating landmarks on a map), path route knowledge (sequential information about relations between landmarks, e.g., directional decisions at intersection), and path survey knowledge (static information about spatial relations between landmarks, e.g., distance comparisons between different sets of landmarks).

Table 2 shows for each article each navigation domain that was included in the measures. Per domain, the findings are marked with = if performance was equal to control participants’ performance without DTD and with ↓ when performance was significantly below the control participants’ performance. The table indicates that most articles include landmark, allocentric location, path route, and path survey measures. Egocentric location knowledge is omitted in most studies. Furthermore, there are only three articles that include measures from all five domains. Landmark knowledge is intact in most cases, whereas people with DTD show impairment in most articles assessing allocentric location knowledge and path survey knowledge. For path route knowledge, the findings appear mixed, with seven instances of impaired performance, one of which only concerned slower responses, and four of intact performance. Strikingly, three out of the four articles indicating impaired landmark knowledge (Iaria et al., 2014; Piccardi et al., 2019; Burles et al., 2020) are the only articles that also report impaired face recognition, except for one (Rusconi et al., 2021).

**Table 2:** Type of assessment included, split up by behavioral, neuroimaging and questionnaire forms of assessment, and navigation domains included. X indicates the type of assessment included, ↓ indicates lower performance than controls, = indicates comparable performance to controls, blank cells indicate the measure was not included. As more than one measure was sometimes used for a navigation domain, mixed findings (↓/=) were possible

Article	Measures included			Navigation domains				
	Behavioral	Neuroimaging	Questionnaire	Landmark	Location egocentric	Location allocentric	Path route	Path survey
Iaria et al., 2009	X	X	X	=		↓	=	↓
Bianchini et al., 2010	X	X	X	=			↓	↓
Iaria & Barton, 2010	X		X	↓	↓/=	↓	↓	↓
Iaria et al., 2014	X	X	X	↓	↓	↓	↓	↓
Palermo et al., 2014a, 2014b	X	X	X	=			↓	=
Palermo et al., 2014a, 2014b	X	X	X	=		↓	↓	↓
Bianchini et al., 2014	X	X		=		↓	↓	↓
Nemmi et al., 2015	X	X	X	=			=	
Barclay et al., 2016	X		X			↓		
Conson et al., 2018	X	X		=	↓	=	=	
Piccardi et al., 2019	X			↓		=	=	=
Burles & Iaria, 2020			X	↓/=		↓		↓
Rusconi et al., 2021	X			=	=	↓	↓	↓
Piccardi et al., 2022a			X				↓	↓
Piccardi et al., 2022b			X					↓

O*bservations.*

The examination of all articles led to a few additional notable observations. DTD is considered a “disorder” by all articles. In contrast, aphantasia, which shares some key characteristics with DTD concerning the extent to which visuospatial information can be mentally visualized, is considered an atypical phenomenon rather than a form of impairment (e.g. Blomkvist, 2023; Dance et al., 2022). Moreover, seven of the articles use the term “patient” to identify an individual with DTD, whereas eight do not use this term. In relation to stigmatization, this could be a meaningful qualification. Lastly, it is worth noting that almost all papers concerning DTD have been produced by two research groups, five of which by the team of Iaria at the University of Calgary, and another nine from the Sapienza Universitá di Roma. Additionally, after its initial discovery, articles have been published at a steady rate of 0–2 per year, with an exception for 2014 when four papers appeared.

## Discussion

The aim of the current literature review was to shed more light on the precise behavioral characteristics of DTD by examining existing current theoretical and empirical evidence. This approach consisted of the analysis of three elements: existing descriptions and criteria for DTD, tests used and performance on the different navigation domains, and observations that can be made in the literature currently available. This information will hopefully stimulate further work on DTD and provide a foundation for future solutions focusing on objective, behavioral assessment, and intervention opportunities in DTD.

The results indicate that the general description of DTD has stabilized in 2014, with four criteria that have been presented. These criteria focus on getting lost frequently throughout life that cannot be explained by cognitive or medical conditions. It could be argued that the steep recent increase in the availability of navigation support systems could make the criterion of frequently getting lost very difficult to implement, as some but not all people with DTD indicate they rely heavily on their smartphones when they navigate. Moreover, lack of specification of “frequently” limits the clarity of this criterion and consequently the possibility to objectively assess it. Furthermore, the nature of getting lost is not specified. Whereas earlier work specifically mentions mental map quality as a key marker, more recently, it has been suggested that DTD could serve as an umbrella term for a range of navigation impairments, including landmark agnosia. We would like to argue that placing all types of navigation problems under the label of DTD, increasing the risk of a jingle fallacy, may actually be harmful in working towards a solution, as it intentionally creates a more diffuse picture of the impairment. As we have seen for navigation impairment in acquired brain injury, a clear behavioral classification of navigation ability is a constructive approach towards a formal diagnostic process and consequent treatment options.

The behavioral patterns resulting from our search show that impairment in DTD is specifically linked to impaired allocentric location knowledge and path survey knowledge. Path route knowledge is impaired in a substantial number of reports but has also been found to be intact in some cases. Furthermore, we observe that landmark knowledge is largely intact, which supports the view that DTD is a selective and thus specific form of navigation impairment. This is further supported by the fact that the rare observations of impairment of landmark knowledge largely overlap with problematic face perception. In three out of four articles in which landmark agnosia is found, prosopagnosia or impaired face imagery is also detected. This could suggest that rather than DTD, a more general form of visual agnosia might be present in these cases that results in several behavioral issues, including getting lost. One of these three articles (Iaria et al., 2014) includes a very large sample size (*N* = 1211) and data reflects the mean performance of the entire sample. It would be very informative to examine the correlation between landmark knowledge and face imagery performance. Rusconi and colleagues (2021) are the only ones reporting a face recognition deficit in absence of landmark knowledge impairment. It should be noted that in this particular case, landmark knowledge was only assessed by verifying familiarity with hometown and European landmarks, so fully separated from memorizing a specific route or environment. This observation concerning a potentially more general visual agnosia or imagery impairment further stimulates the development of a more clearly defined definition of DTD. Lastly, egocentric location knowledge (e.g., observer-based perspective) is rarely included and could thus provide additional refinement to the cognitive characteristics of DTD. Combined, this overview shows that more standardization in assessment would be helpful in the accumulation and comparison of DTD navigation performance data.

Additionally, we observed that DTD is considered a disorder by all authors, and people with DTD are labeled as “patients” in a substantial number of publications. Using the term “patient” could unintentionally enhance stigmatization, rather than reducing it. Surprisingly, since the initial reports, DTD has mainly been described by two research groups. This could reflect the limited awareness of DTD in the scientific community, the general population and people with DTD themselves; increasing this awareness could stimulate more detailed and clinically oriented research in this population.

To conclude, more cognitive specification of DTD appears appropriate and helpful to resolve the jingle fallacy that is likely present, which in turn will provide the foundation for future objective assessment methods. The spatial impairment in DTD appears to be isolated to mental map quality, as used in allocentric location and path survey knowledge, as well as route knowledge to some extent. Landmark knowledge remains largely unaffected in DTD, except for when face agnosia is present as well. We suggest separating those instances from cases of pure DTD to eliminate a jingle fallacy and to provide clarity in the diagnostic process and subsequent intervention options. The current findings suggest that in future work, a comprehensive assessment of navigation ability should be included, and to specifically assess egocentric location knowledge as well. During assessment, exclusion of other conditions that could have comparable consequences concerning getting lost, such as visual agnosias and landmark agnosia, is advisable as well. Also, a focus on identification of DTD at an earlier age would be beneficial to support young people with DTD at the age when they start developing independence and their lifelong navigation strategies.

## Data Availability

Data will be made available on DataVerseNL upon publication.

## References

[CR1] Barclay, S. F., Burles, F., Potocki, K., Rancourt, K. M., Nicolson, M. L., Bech-Hansen, N. T., & Iaria, G. (2016). Familial aggregation in developmental topographical disorientation (DTD). *Cognitive Neuropsychology,**33*(7–8), 388–397. 27923326 10.1080/02643294.2016.1262835

[CR2] Bianchini, F., Incoccia, C., Palermo, L., Piccardi, L., Zompanti, L., Sabatini, U., ... & Guariglia, C. (2010). Developmental topographical disorientation in a healthy subject. Neuropsychologia, 48(6), 1563–1573. 10.1016/j.neuropsychologia.2010.01.025 20144632

[CR3] Bianchini, F., Palermo, L., Piccardi, L., Incoccia, C., Nemmi, F., Sabatini, U., & Guariglia, C. (2014). Where am I? A new case of developmental topographical disorientation. *Journal of Neuropsychology,**8*(1), 107–124. 23336564 10.1111/jnp.12007

[CR4] Blomkvist, A. (2023). Aphantasia: In search of a theory. *Mind & Language,**38*(3), 866–888.

[CR5] Burgess, N. (2006). Spatial memory: How egocentric and allocentric combine. *Trends in Cognitive Sciences,**10*(12), 551–557. 17071127 10.1016/j.tics.2006.10.005

[CR6] Burles, F., & Iaria, G. (2020). Behavioural and cognitive mechanisms of developmental topographical disorientation. *Scientific Reports,**10*(1), 20932. 33262419 10.1038/s41598-020-77759-8 PMC7708628

[CR7] Chrastil, E. R., & Warren, W. H. (2013). Active and passive spatial learning in human navigation: Acquisition of survey knowledge. *Journal of Experimental Psychology: Learning, Memory, and Cognition,**39*(5), 1520. 23565781 10.1037/a0032382

[CR8] Claessen, M. H., & van der Ham, I. J. (2017). Classification of navigation impairment: A systematic review of neuropsychological case studies. *Neuroscience & Biobehavioral Reviews,**73*, 81–97. 27993606 10.1016/j.neubiorev.2016.12.015

[CR9] Conson, M., Bianchini, F., Quarantelli, M., Boccia, M., Salzano, S., Di Vita, A., & Guariglia, C. (2018). Selective map-following navigation deficit: A new case of developmental topographical disorientation. *Journal of Clinical and Experimental Neuropsychology,**40*(9), 940–950. 29614925 10.1080/13803395.2018.1451493

[CR10] Dance, C. J., Ipser, A., & Simner, J. (2022). The prevalence of aphantasia (imagery weakness) in the general population. *Consciousness and Cognition,**97*, 103243. 34872033 10.1016/j.concog.2021.103243

[CR11] Denis, M., & Loomis, J. M. (2007). Perspectives on human spatial cognition: Memory, navigation, and environmental learning. *Psychological Research Psychologische Forschung,**71*, 235–239.

[CR12] Epstein, R. A., Patai, E. Z., Julian, J. B., & Spiers, H. J. (2017). The cognitive map in humans: Spatial navigation and beyond. *Nature Neuroscience,**20*(11), 1504–1513. 29073650 10.1038/nn.4656 PMC6028313

[CR13] Fragueiro, A., Cury, C., Santacroce, F., Burles, F., Iaria, G., & Committeri, G. (2024). Medial positioning of the hippocampus and hippocampal fissure volume in Developmental Topographical Disorientation. *Hippocampus,**34*(4), 204–216. 38214182 10.1002/hipo.23599

[CR14] Hanfstingl, B., Oberleiter, S., Pietschnig, J., Tran, U. S., & Voracek, M. (2024). Detecting jingle and jangle fallacies by identifying consistencies and variabilities in study specifications–A call for research. *Frontiers in Psychology,**15*, 1404060. 39282677 10.3389/fpsyg.2024.1404060 PMC11393684

[CR15] Iaria, G. (2013). Developmental topographical disorientation: Lost every day. *The Lancet Neurology,**12*(8), 745.

[CR16] Iaria, G., Arnold, A. E., Burles, F., Liu, I., Slone, E., Barclay, S., ... & Levy, R. M. (2014). Developmental topographical disorientation and decreased hippocampal functional connectivity. Hippocampus, 24(11), 1364–1374. 10.1002/hipo.22317 24976168

[CR17] Iaria, G., & Barton, J. J. (2010). Developmental topographical disorientation: A newly discovered cognitive disorder. *Experimental Brain Research,**206*, 189–196. 20431873 10.1007/s00221-010-2256-9

[CR18] Iaria, G., Bogod, N., Fox, C. J., & Barton, J. J. (2009). Developmental topographical disorientation: Case one. *Neuropsychologia,**47*(1), 30–40. 18793658 10.1016/j.neuropsychologia.2008.08.021

[CR19] Iaria, G., & Burles, F. (2016). Developmental topographical disorientation. *Trends in Cognitive Sciences,**20*(10), 720–722. 27450709 10.1016/j.tics.2016.07.004

[CR20] Kim, J. G., Aminoff, E. M., Kastner, S., & Behrmann, M. (2015). A neural basis for developmental topographic disorientation. *Journal of Neuroscience,**35*(37), 12954–12969. 26377479 10.1523/JNEUROSCI.0640-15.2015 PMC4571612

[CR21] Klatzky, R. L. (1998). Allocentric and egocentric spatial representations: Definitions, distinctions, and interconnections. In Spatial cognition: An interdisciplinary approach to representing and processing spatial knowledge (pp. 1–17). Berlin, Heidelberg: Springer Berlin Heidelberg.

[CR22] Nemmi, F., Bianchini, F., Piras, F., Péran, P., Palermo, L., Piccardi, L., ... & Guariglia, C. (2015). Finding my own way: An fMRI single case study of a subject with developmental topographical disorientation. Neurocase, 21(5), 573–583. 10.1080/13554794.2014.960424 25279725

[CR23] Palermo, L., Foti, F., Ferlazzo, F., Guariglia, C., & Petrosini, L. (2014a). I find my way in a maze but not in my own territory! Navigational processing in developmental topographical disorientation. *Neuropsychology,**28*(1), 135. 24219605 10.1037/neu0000021

[CR24] Palermo, L., Piccardi, L., Bianchini, F., Nemmi, F., Giorgio, V., Incoccia, C., ... & Guariglia, C. (2014). Looking for the compass in a case of developmental topographical disorientation: A behavioral and neuroimaging study. Journal of Clinical and Experimental Neuropsychology, 36(5), 464–481. 10.1080/13803395.2014.904843 24742227

[CR25] Piccardi, L., De Luca, M., Di Vita, A., Palermo, L., Tanzilli, A., Dacquino, C., & Pizzamiglio, M. R. (2019). Evidence of taxonomy for developmental topographical disorientation: Developmental landmark agnosia case 1. *Applied Neuropsychology: Child,**8*(2), 187–198. 29192795 10.1080/21622965.2017.1401477

[CR26] Piccardi, L., Palmiero, M., Cofini, V., Verde, P., Boccia, M., Palermo, L., ... & Nori, R. (2022a). “Where am I?” A snapshot of the developmental topographical disorientation among young Italian adults. PLoS One, 17(7), e0271334. 10.1371/journal.pone.0271334 PMC9299294 35857777

[CR27] Piccardi, L., Cofini, V., Palmiero, M., Verde, P., Boccia, M., Palermo, L., ... & Nori, R. (2022b). Where am I? Searching for the tangle in the developmental topographical disorientation. Neurology International, 14(4), 824–838. 10.3390/neurolint14040067 PMC9589977 36278691

[CR28] Riddick, B. (2000). An examination of the relationship between labelling and stigmatisation with special reference to dyslexia. *Disability & Society,**15*(4), 653–667.

[CR29] Rusconi, M. L., Fusi, G., Stampatori, C., Suardi, A., Pinardi, C., Ambrosi, C., ... & Mattioli, F. (2021). Developmental topographical disorientation with concurrent face recognition deficit: A case report. Frontiers in Psychiatry, 12, 654071. 10.3389/fpsyt.2021.654071 PMC8267524 34248701

[CR30] Shaw, S. C. K., & Anderson, J. L. (2017). Doctors with dyslexia: A world of stigma, stonewalling and silence, still? *MedEdPublish,**6*, 29.

[CR31] Spiers, H. J., Coutrot, A., & Hornberger, M. (2023). Explaining world-wide variation in navigation ability from millions of people: Citizen science project sea hero quest. *Topics in Cognitive Science,**15*(1), 120–138. 34878689 10.1111/tops.12590

[CR32] Stoeber, J., & Rountree, M. L. (2021). Perfectionism, self-stigma, and coping in students with dyslexia: The central role of perfectionistic self-presentation. *Dyslexia,**27*(1), 62–78. 32803909 10.1002/dys.1666

[CR33] Thornberry, C., Cimadevilla, J. M., & Commins, S. (2021). Virtual Morris water maze: Opportunities and challenges. *Reviews in the Neurosciences,**32*(8), 887–903. 33838098 10.1515/revneuro-2020-0149

[CR34] Thorndike, E. L. (1904). *An introduction to the theory of mental and social measurements*. Teachers College, Columbia University.

[CR35] van der Ham, I. J., & Claessen, M. H. (2020). How age relates to spatial navigation performance: Functional and methodological considerations. *Ageing Research Reviews,**58*, 101020. 31954190 10.1016/j.arr.2020.101020

[CR36] van der Ham, I. J., & Claessen, M. H. (2022). A clinical guide to assessment of navigation impairment: Standardized subjective and objective instruments and normative data. *Journal of Clinical and Experimental Neuropsychology,**44*(7), 487–498. 36129157 10.1080/13803395.2022.2123895

[CR37] van der Ham, I. J., Claessen, M. H., Evers, A. W., & van der Kuil, M. N. (2020). Large-scale assessment of human navigation ability across the lifespan. *Scientific Reports,**10*(1), 3299. 32094394 10.1038/s41598-020-60302-0 PMC7039892

[CR38] Van der Ham, I. J., Kant, N., Postma, A., & Visser-Meily, J. M. (2013). Is navigation ability a problem in mild stroke patients? Insights from self-reported navigation measures. *Journal of Rehabilitation Medicine,**45*(5), 429–433. 23615778 10.2340/16501977-1139

[CR39] Van der Kuil, M. N. A., Visser-Meily, J. M. A., Evers, A. W. M., & van der Ham, I. J. M. (2022). Navigation ability in patients with acquired brain injury: A population-wide online study. *Neuropsychological Rehabilitation,**32*(7), 1405–1428. 33715586 10.1080/09602011.2021.1893192

[CR40] Wang, R. F., & Spelke, E. S. (2000). Updating egocentric representations in human navigation. *Cognition,**77*(3), 215–250. 11018510 10.1016/s0010-0277(00)00105-0

[CR41] Weisberg, S. M., Schinazi, V. R., Newcombe, N. S., Shipley, T. F., & Epstein, R. A. (2014). Variations in cognitive maps: Understanding individual differences in navigation. *Journal of Experimental Psychology: Learning, Memory, and Cognition,**40*(3), 669–682. 24364725 10.1037/a0035261

[CR42] Wolbers, T., Weiller, C., & Büchel, C. (2004). Neural foundations of emerging route knowledge in complex spatial environments. *Cognitive Brain Research,**21*(3), 401–411. 15511655 10.1016/j.cogbrainres.2004.06.013

